# Screening Gene Expression-Related Alternative Splicing Event Signature for Colon Cancer Prognostic Prediction

**DOI:** 10.1155/2022/9952438

**Published:** 2022-01-27

**Authors:** Jie Xu, Jingfeng Chen, Chuan Cheng, Huixia Li, Peng Gao, Jiujian Zheng, Jianping Wang

**Affiliations:** Department of Anorectal Surgery, Lishui Municipal Central Hospital, Lishui Hospital, Zhejiang University School of Medicine, Lishui, Zhejiang Province, China

## Abstract

Colon cancer is a kind of common intestinal disease, and early diagnosis of colon cancer is crucial for patient's prognosis. RNA alternative splicing (AS) is an RNA modification that affects cancer occurrence. RNA AS detection is promising to improve the in-depth understanding of the pathological mechanisms in colon cancer. In this study, differential analysis was performed to determine colon cancer-related AS events and the corresponding parental genes. Subsequently, GO functional annotation analysis was carried out on the parental genes, which revealed that these AS events might affect cell adhesion and cell growth. Besides, protein-protein interaction (PPI) network was established with the parental genes, in which MCODE was utilized to identify major functional modules. Enrichment analysis for the major functional module was implemented again, which demonstrated that these genes were mainly concentrated in the ribosome, protein ubiquitination, cell adhesion molecule binding, and other relevant biological functions. Next, differentially expressed genes (DEGs) were screened from colon cancer and normal tissues and overlapped with the parental genes, by which 55 gene expression-associated AS and the corresponding 45 genes were obtained. Moreover, a correlation analysis between splicing factors (SFs) and AS was done to identify interactions. On this basis, an SF-AS network was constructed. The univariate Cox regression analysis was employed to screen prognostic AS signature and establish a risk model. To assess the model, K-M and ROC analyses were done for model assessment, indicating the effective prediction performance. Combined with common clinicopathological features, the multivariate Cox regression analysis was conducted to confirm whether the risk model could be considered as an independent prognostic indicator. Finally, the expression status of the parental genes for the prognostic AS was evaluated between normal and colon cancer cells using qRT-PCR. In summary, TCGA SpliceSeq data were comprehensively analyzed, and a 5-AS prognostic model was constructed for colon cancer.

## 1. Introduction

Colon cancer is a highly prevalent type of cancer. In 2018, there were about 1,090,000 new cases of colon cancer and 551,000 colon cancer deaths worldwide, causing a great loss to the whole society [1]. The risk factors leading to colon cancer initiation include unhealthy diet and lifestyle, long-term use of specific drugs, genetic factors, and chronic intestinal inflammation [2]. The unhealthy diet and lifestyle mainly refer to long-term smoking, excessive drinking, and long-term consumption of foods containing carcinogens such as smoked and salted meat [3]. Early diagnosis and precise prognostic assessment for colon cancer patients are therefore the best way to reduce colon cancer deaths. Several factors can be used to predict patients' prognostic status, in which clinicopathological features such as TMN stage, age, and gender were commonly considered as traditional prognostic factors [4], but traditional factors are not sufficient for precise prognostic prediction, which is the reason why we designed our analysis for screening a prognostic signature.

Gene signature screening is a new avenue to determine a set of genes for prognostic risk prediction. Researchers have reported multiple gene biomarkers, which are capable of assessing the prognostic risk of colon cancer patients [5], including TP53, APC, and KARS [6]. At present, numerous studies focus on screening biomarkers based on mRNA expression, DNA methylation, and other data, and some of the results are successfully applied in clinical practice. With the rapidly emerging interests in RNA alternative splicing (AS) research, there are studies focusing on the changes in RNA AS events in colon cancer patients [7], and some researchers regarded AS signatures as potential prognostic biomarkers for various tumors [8–10].

RNA AS event is an epigenetic RNA post-transcriptional modification by the spliceosome, which can affect the translation of RNA protein [11]. So far, many studies reveal the effects of RNA AS events on cancer in the views of molecular mechanism and cell behaviors. For example, the AS events of the BHC80 gene can stimulate tumor proliferation through the MyD88-p38-TTP pathway and thus promote the malignant progression of prostate cancer [12]. Another study has indicated that SF3B2 can affect the resistance of prostate cancer to AR targeting therapy by affecting the AS events of the AR gene [13]. Moreover, it has been reported that AS can affect gene expression, thereby resulting in cancer progression regulation. For example, DEAD-box helicase 56 can be regulated by AS events, thus affecting the colon cancer process [14]. Considering the studies mentioned above, it is expected that AS-induced gene expression dysregulation may play an important role in cancer occurrence and development. Therefore, gene expression-associated AS signature could be a promising indicator for cancer prognosis.

In this study, colon cancer-related expression data from The Cancer Genome Atlas (TCGA) database were accessed to analyze the AS status in colon cancer patients by the TCGA SpliceSeq database. Five AS events, significantly related to the prognosis, were screened by univariate analysis, and the prediction efficiency and independence of the prognostic signature were assessed. In conclusion, our findings may contribute to a deep understanding of pathological mechanisms of colon cancer and the development of colon cancer prognostic biomarkers.

## 2. Materials and Methods

### 2.1. Data Downloading

**Figure 1:**
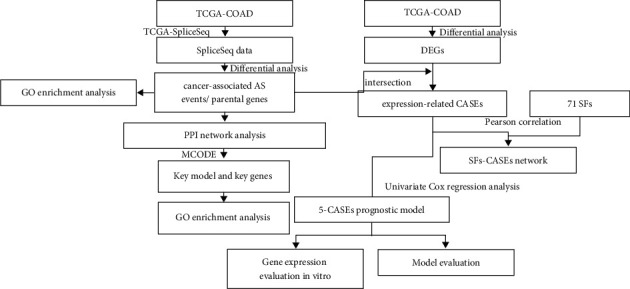
Workflow of this study.

The mRNA expression data and corresponding clinical information of patients with colon cancer were downloaded from the TCGA database (https://portal.gdc.cancer.gov/), followed by data preprocessing, from which we obtained 41 normal tissue samples and 462 tumor samples for subsequent analyses (excluding formalin-soaked samples). The samples were divided into the training and validation sets in the ratio of 7 to 3. The data downloading time was May 9, 2020. Colon cancer AS data were obtained from the TCGA SpliceSeq database (https://bioinformatics.mdanderson.org/TCGASpliceSeq/). The samples with a percent spliced-in (PSI) value ranking the top 75% were selected for subsequent analyses. A total of 41 normal tissue samples and 458 tumor samples were collected. The flowchart of this study (Figure 1) was provided to clarify the analysis process of this study.

### 2.2. Identification of Cancer-Associated AS Events

To determine cancer-associated AS events (CASEs) in colon cancer, differential PSI analysis for AS events between cancer tissue and para-cancerous tissue was conducted, followed by the *t*-test. In detail, cancer tissue samples with matched normal tissue samples were screened, by which 41 pairs of tissue samples were selected, and PSI differential analysis was followed up for determining CASEs. AS events with |log2FC| ≥ 1 and FDR < 0.05 were selected as CASEs. Subsequently, GO annotation analysis was performed on the corresponding parental genes using R package *clusterProfiler*. The biological functions with *p* value <0.05 were significantly enriched ones.

### 2.3. PPI Network Construction and Analysis of CASE Parental Genes

Firstly, the PPI of the CASE parental genes was calculated by the STRING database (https://www.string-db.org/). A PPI network was constructed with confidence >0.9 as the threshold value, and Cytoscape software (v3.7.2) was used for visualization. Then, the MCODE plug-in was implemented to identify the key genes and module. After the key genes were obtained, the R package *clusterProfiler* was used for GO functional annotation analysis and biological functions with significant enrichment were screened with *p* < 0.05.

### 2.4. CASE Network Construction Combined with Splicing Factors (SFs)

According to the mRNA expression data of TCGA-COAD, differential expression analysis was conducted using *R* package edge*R*, and genes with |log2FC| > 2 and FDR < 0.05 were selected as differentially expressed genes (DEGs). The intersection of DEGs and the parental genes was referred to as expression-related parental genes. Subsequently, 71 splicing factors (SFs) from the literature review were selected for correlation analysis with the CASEs, which matched the intersected genes, and then, a SF-CASE network was constructed (Table S1).

### 2.5. Survival Analysis

The CASEs obtained from the above studies were analyzed by the univariate Cox regression analysis via the “survival” package to screen AS events significantly related to prognosis in the training set. Using the “survival” package, the ROC analysis was conducted both in the training and validation sets. The K-M analysis was followed up in the validation set. The univariate and multivariate Cox regression analyses on the model risk score and common clinicopathological features were carried out in the training set. The next nomogram on the multivariate Cox regression was performed, after which calibration curves were introduced for evaluating the prediction.

### 2.6. Cell Culture

Colonic cell line CCD-18Co and colon cancer cell line HT29 were purchased from Bena Culture Collection (Beijing, China). The cells were cultured in RPMI 1640 (Thermo Fisher Scientific, Massachusetts, USA) with 10% FBS (Thermo Fisher Scientific, Massachusetts, USA) and maintained at 37°C, 5% CO_2._

### 2.7. Real-Time PCR

Total RNA was extracted from cells using TRIzol (Thermo Fisher Scientific, Massachusetts, USA), and the cDNA was synthesized by PrimeScript RT Reagent Kit (Takara Bio, Kyoto, Japan). Based on the collected cDNA, real-time PCR was introduced with SYBR Premix Ex Taq II (Takara Bio, Kyoto, Japan) on QuantGene 9600 Real-Time PCR System (Hangzhou, China), and the primers were presented as follows (Table 1). *β*-actin was selected as the internal reference gene, and the PCR results were calculated by the 2^−ΔΔCT^ method.

### 2.8. Result Statistics

Each experiment was repeated three times, and the experimental results were performed with mean value ± standard deviation. Differential significance was tested by the *t*-test. The statistical plot was presented using GraphPad Prism (GraphPad Software, California, USA).

## 3. Results

### 3.1. AS Status for Colon Cancer Patients

**Figure 2:**
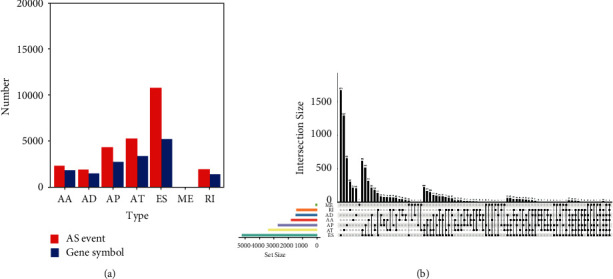
Overview of AS events in colon cancer of TCGA SpliceSeq. (a) Number of AS events and corresponding parental genes of colon cancer patients in TCGA SpliceSeq. The red bar represents AS events in different types, and the blue bar represents parental genes in different types of AS events; (b) the UpSet plot of AS events. The strips on the left side presented subsets of 7 different types of AS events. Dots without connection presented the single type of AS event, and dots connected by lines showed multiple types of AS occurring simultaneously. The histogram presented subsets with single or multiple AS event occurrence.

Colon cancer-related AS data were extracted by TCGA SpliceSeq, including 35,391 AS events and the corresponding 9,085 parental genes. AS events with an average PSI higher than 0.05 were screened, whereby 26,618 AS events and the corresponding 8,871 parental genes were finally determined. These AS events could be divided into 7 different AS modes: alternate terminator (AT), retained intron (RI), alternate acceptor (AA) site, alternate promoter (AP), exon skip (ES), alternate donor (AD) site, and mutually exclusive exon (ME). According to the type of the AS events, statistical analysis for AS events was conducted with the UpSet plot of AS events (Figures 2(a) and 2(b)). Above all, ES occurs most frequently among the 7 AS modes.

### 3.2. Identification of CASEs in Colon Cancer

**Figure 3:**
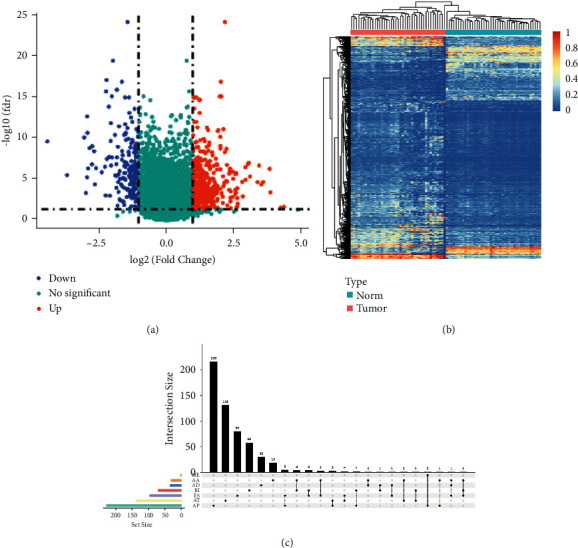
Overview of CASEs. (a) Volcano plot of differential CASEs in TCGA-COAD cohort; (b) heat map of CASEs in 41 pairs of colon cancer tissue and para-cancerous tissue. The row represents different tissue types, and the column represents different CASEs. Blue means low expression, and red means high expression; (c) UpSet plot shows different splicing modes of CASEs. The strap in the lower left corner shows the number of AS events contained in each AS type. The dots and lines in the lower right corner indicate different AS events. The histogram above the abscissa represents the number of events in the intersection.

To identify CASEs, the cancer tissue was paired with the para-cancerous tissue, and differential analysis on PSI between the paired groups was conducted, where 669 differential AS events were screened. Differential AS events were considered as CASEs (Figure 3(a)). Based on the CASEs, the heat map and UpSet plot were plotted (Figures 3(b) and 3(c)). In conclusion, the difference in PSI for the CASEs between tumor and normal tissues was visibly presented.

### 3.3. Functional Enrichment Analysis

**Figure 4:**
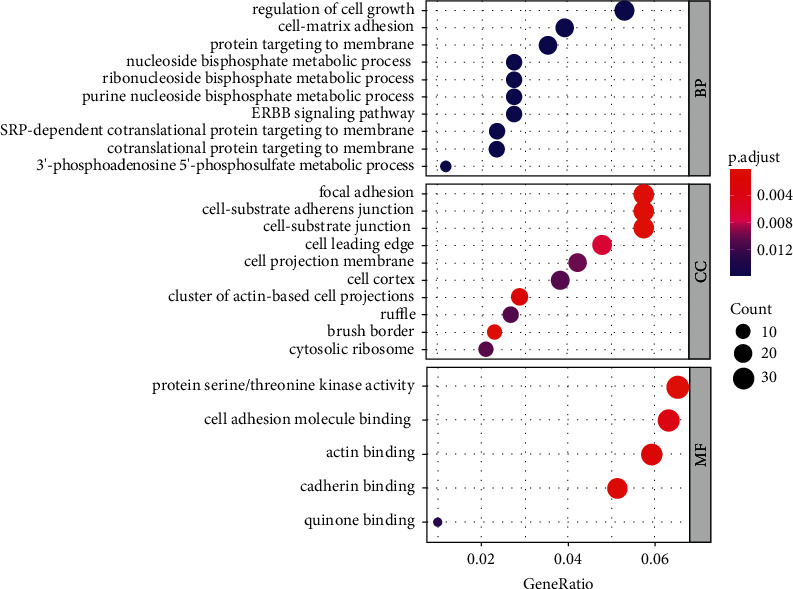
GO annotation result of parental genes of CASEs.

Since we identified CASEs of colon cancer, parental genes of these CASEs were analyzed in terms of biological functions via GO enrichment analysis. The results presented that these parental genes were mainly enriched in biological processes (such as regulation of cell growth and cell-matrix expression), cellular components (such as focal adhesion and cell-substrate junction), and molecular functions (such as cell adhesion molecule binding and actin binding) (Figure 4). The results suggested that these AS events may affect cell adhesion and cell growth.

### 3.4. PPI Network

**Figure 5:**
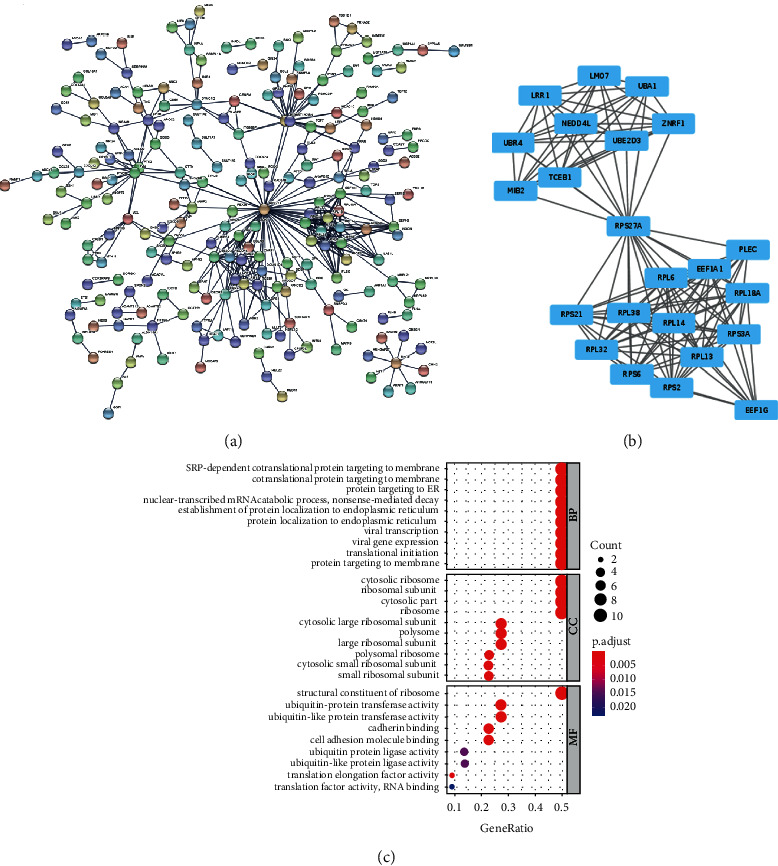
PPI network construction and analysis of CASE parental genes. (a) PPI network constructed according to the parental genes of the selected CASEs; (b) key genes screened by MCODE; (c) GO annotation result of the 23 key genes.

To further understand the interactions and major functions of the parental genes, a PPI network for the parental genes was generated (Figure 5(a)). Subsequently, the MCODE plug-in was applied to screen key genes and key functional module, and then, a module with 23 key genes was obtained (Figure 5(b)). The GO functional annotation enrichment analysis of these 23 genes suggested that these genes were mainly enriched in ribosome, protein ubiquitination, cell adhesion molecule binding, and other relevant biological functions (Figure 5(c)).

### 3.5. Screening Expression-Related Parental Genes

**Figure 6:**
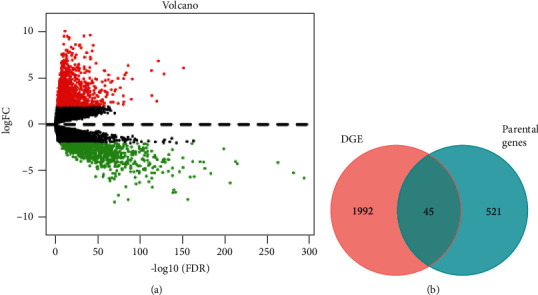
Screening of CASEs related to DEGs. (a) Volcano plot of differentially expressed mRNAs; red dots represent upregulated genes, and blue dots represent downregulated genes; (b) the Venn diagram plotted to DEGs and the parental genes of CASEs.

To identify the parental genes whose expression levels were changed by AS events, the differential mRNA expression analysis for tumor and normal tissues was conducted firstly, where 1,098 upregulated and 939 downregulated genes were screened (Figure 6(a)). Then, the DEGs were intersected with the parental genes obtained above, by which 45 expression-related parental genes were obtained (Figure 6(b)). 55 AS events, referred to as expression-related CASEs, occurring in these genes were selected as well.

**Figure 7:**
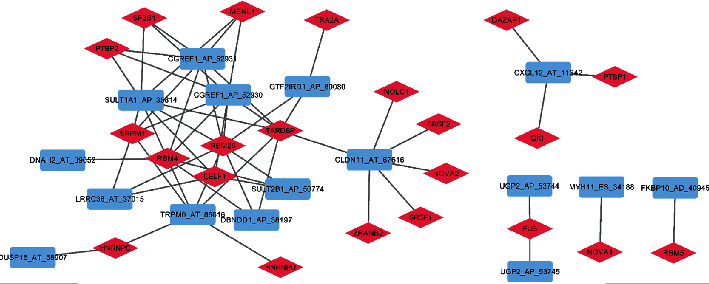
Network of CASEs and SFs.

Since SFs play a crucial role in AS regulating, their expression level may also play an important role in the AS regulation. Introducing the Pearson correlation analysis, we selected the significantly correlated pairs to construct SF-CASE network based on the 71 SFs from the literature review and the 55 expression-related CASEs, where 16 expression-related CASEs and 22 SFs were included (Figure 7).

### 3.6. Mining and Evaluating Prognosis-Related CASEs

**Figure 8:**
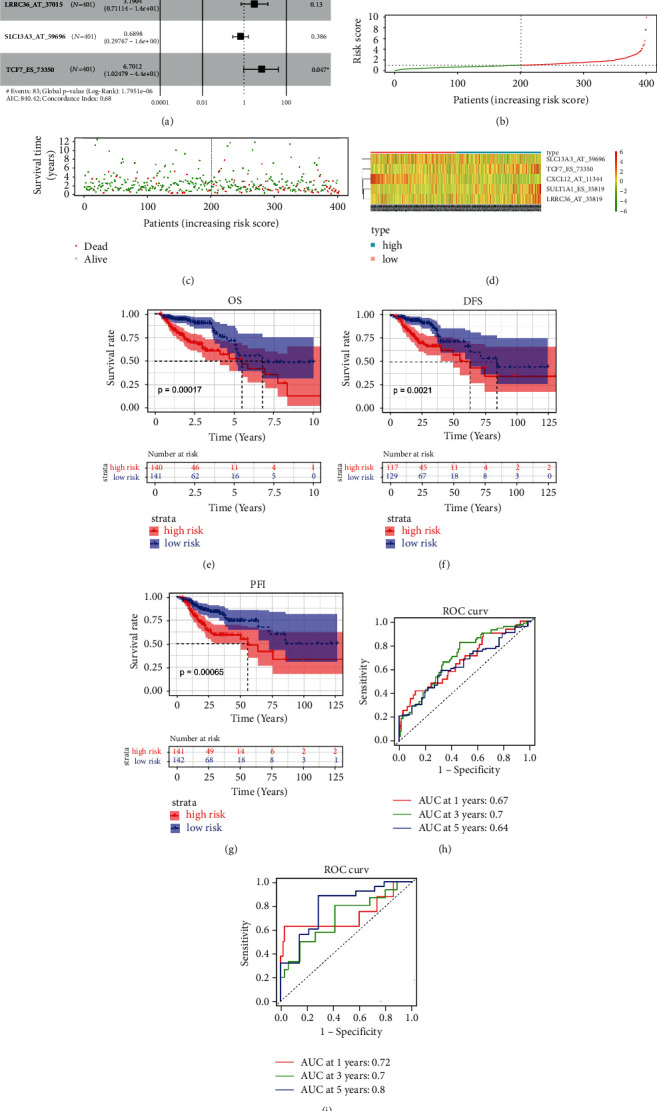
Screening and validation of AS events related to the prognosis. (a) Univariate Cox regression result shows the 5 prognosis-related CASEs; (b) PSI heat map of the 5 key CASEs in the high- and low-risk score groups; (c, d) survival status distribution of samples with different risk scores; (e–g) survival analyses between the high- and low-risk score groups on OS, PFI, and DFS, respectively; (h, i) ROC curves for the 5-CASE signature in the training and validation sets, respectively.

**Figure 9:**
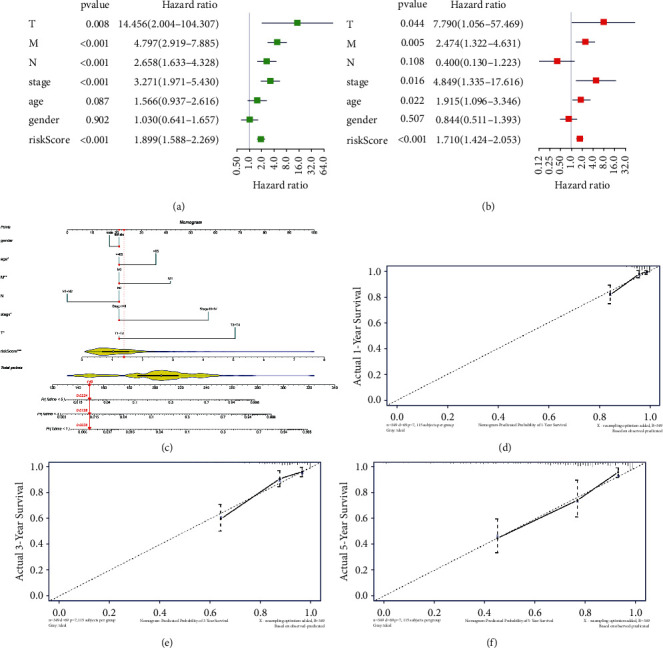
Assessment of the prognostic model. (a, b) Univariate and multivariate Cox regression for common clinicopathological features and risk scores; (c) nomogram for common clinicopathological features and risk scores; (d–f) calibration curves for 1-, 3-, 5-year survival, respectively.

To understand the effects of the expression-related CASEs on patient's prognostic status, the univariate Cox analysis was conducted combined with patient survival information and 5 CASEs with significant prognostic correlation were obtained. Among them, the hazard ratios (HRs) of SULT1A1_ES_35819, LRRC36_AT_37015, and TCF7_ES_73350 were greater than 1; thus, they were risk factors. The HRs of CXCL12_AT_11344 and SLC13A3_AT_59696 were less than 1; thus, they were protective factors. Finally, we generated a 5-CASE prognostic model (Table S2, Figure 8(a)). The risk score of colon cancer in the TCGA data set was calculated, and with the median risk score as the cutoff value, patients were classified into the high- and low-risk groups (Figure 8(b)). A scatter plot of the patient's survival status was drawn. The results illustrated that with the increase in risk score, the number of deaths increased and survival time decreased (Figure 8(c)). Meanwhile, a heat map of expression of 5 CASEs in the high- and low-risk groups was plotted. The heat map depicted that as the risk score increased, the expression levels of risk factors such as SULT1A1_ES_35819, LRRC36_AT_37015, and TCF7_ES_73350 were gradually increased, while the expression levels of protective factors such as CXCL12_AT_11344 and SLC13A3_AT_59696 were gradually decreased (Figure 8(d)). Then, we plotted the survival curves of OS, PFI, and RFS in the high- and low-risk groups. As depicted in Figures 8(e)–8(g), the prognosis of patients in the low-risk group was markedly better than that in the high-risk group. Finally, ROC curves of the training set and the validation set illustrated that in the training set, AUC values of 1-, 3-, and 5-year survival predicted by the 5-CASE model were 0.67, 0.7, and 0.64, respectively. In the validation set, AUC values of 1-, 3-, and 5-year survival predicted by the 5-CASE model were 0.72, 0.7, and 0.8, respectively (Figures 8(h) and 8(i)). The above results demonstrated that the 5-CASE model had good performance in predicting patient's prognosis. Furthermore, to assess the independence of the model, the univariate and multivariate Cox regression analyses were introduced based on risk score and the common clinicopathological features. As the result indicated, our prognostic model showed robust independence (Figures 9(a) and 9(b)). Next, a nomogram was plotted for the prediction of survival status combined with the clinicopathological features (Figure 9(c)), followed by evaluation by calibration curve (Figures 9(d)–9(f)), where all the results proved a precise prediction by the nomogram. All above, 5-CASE signature could be effectively used for prognostic prediction.

### 3.7. Evaluating Gene Expression Status

**Figure 10:**
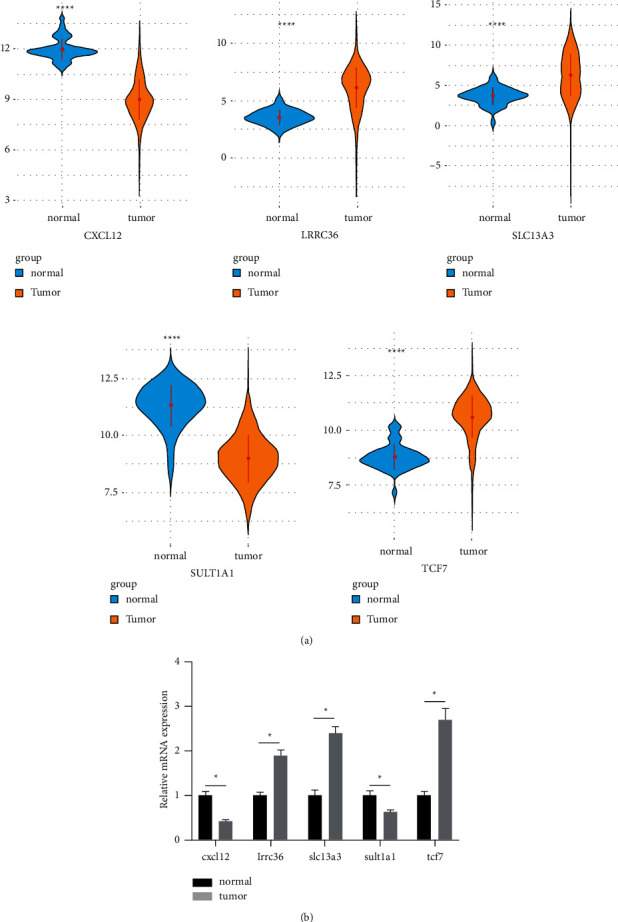
mRNA expression evaluation for the 5 parental genes. (a) mRNA expression status between tumor and normal tissues on the TCGA database; (b) mRNA expression between tumor and normal cell lines. ^*∗*^ means *p* < 0.05, and ^*∗∗∗*^ means *p* < 0.001.

To assess mRNA expression status, we focused on the parental genes of the 5 CASEs, namely CXCL12, SULT1A1, LRRC36, SLC13A3, and TCF. Initially, the expression of the 5 genes in tumor and normal samples was plotted based on TCGA data (Table S3). As the plot illustrated, all the genes were significantly dysregulated in tumor samples (Figure 10(a)). Furthermore, colon cancer and normal cell lines were prepared for mRNA expression analysis. The result showed a consistency with TCGA data to a certain extent (Figure 10(b)). As expected, the 5 genes were abnormally expressed in tumor cell lines, which were possibly caused by CASEs.

## 4. Discussion

As a common cancer, colon cancer causes a large number of deaths in patients every year [15]. Biomarkers are biochemical indicators detected from cancer tissue or blood tissue of patients that can be used to determine the prognosis of patients. The development of colon cancer-related biomarkers can help clinicians better judge the prognostic risk of patients and improve the prognosis of colon cancer patients [16]. With the development of bioinformatics, it has become a hot research field to explore cancer-related biomarkers through AS differences [17]. Hence, we also attempted to explore the relationship between colon cancer incidence and AS by studying AS relevant data of colon cancer. In this study, colon cancer-related SpliceSeq data were analyzed to find relevant biomarkers that affect colon cancer prognosis. The results illustrated that in patients with colon cancer, AS events mainly included ME, AA, AD, RI, ES, AT, and AP, among which ES had the highest frequency. Further analysis of differences in cancer tissues and adjacent tissues disclosed that AP had the highest frequency. The above results demonstrated that ES had a higher frequency in common colon tissue cells, and AP may be biased toward colon cancer tissue. Based on TCGA SpliceSeq data, Xu et al. [18] identified survival-related AS events and features in adrenocortical carcinoma, disclosing that AP events in adrenocortical carcinoma are implicated in survival and have the favorable prognostic ability. Hence, AP events may be pivotal in carcinogenesis.

According to the identified parental genes, we established a PPI network and screened the key parental genes. Among these genes, EEF1A1, PLEC, and TCEB1 are common cancer-related genes. EEF1A1 can promote tumor spread through the STAT1-cyclin D1 pathway [19]. PLEC is a susceptibility gene for testicular cancer [20]. TCEB1 can facilitate cell invasion in prostate cancer and the malignant progression of prostate cancer [21]. These results indicate that the parental genes of AS events are closely related to the occurrence of cancer, and AS events may be an essential avenue to influence cancer occurrence. Through the enrichment analysis of the parental genes of the key AS events we identified, we found the enrichment of these genes mainly presented in the ribosome, protein ubiquitination, cell adhesion molecule binding, and other related biological functions. In addition to the study of these parental genes, the changes in mRNA expression in TCGA-COAD were also analyzed, and 16 differential mRNA-related AS events associated with SF were selected accordingly. SFs are a kind of regulatory factor for AS events, and most members are related to the occurrence of cancer. For example, Tra2-*β* was found to promote the occurrence of cancer by affecting the AS events of CD44 [22]. RBM25, TARDBP, and CELF1 were found to be correlated with many CASEs, among which RBM25 was thought to inhibit the progression of acute myelogenous leukemia by affecting MYC activity [23]. CELF1 can foster the migration and invasion of tumor cells by targeting ETS2 and other genes [24]. Most of the SFs screened out by the above results are related to the occurrence and progression of cancer, and it is believed that the SFs screened out in this study and the corresponding CASEs may be key factors affecting the incidence of colon cancer.

After screening out 55 key CASEs associated with differential mRNA, the univariate regression analysis was performed to screen the CASEs that were significantly related to the prognosis of patients, by which 5-CASE prognostic model was constructed. In this study, it was found that the AT events of CXCL12 may be the major AS events affecting the prognostic risk of colon cancer to a large extent, and patients with higher CXCL12_AT_11344 PSI had better prognoses. SUL1A1 is also believed to be associated with cancer, and existing studies suggest that single nucleotide polymorphisms of SUL1A1 are associated with a variety of cancers, such as endometrial cancer, esophageal cancer, and breast cancer [25–27]. Although the correlation between SUL1A1 and cancer has been fully discussed in the existing studies, the relationship between the AS events of SUL1A1 and cancer has not been pointed out. Here, it was found that the survival time of patients with higher SUL1A1_ES_35819 PSI was significantly shorter. Moreover, the prognostic prediction value of the 5-CASE model was evaluated by a series of methods, such as K-M and ROC analyses, indicating that the model presented the promising ability for prognostic assessment.

In this study, colon cancer-related CESE data were screened, and the corresponding parental genes were selected using various bioinformatic methods as well. Then, combined with the differential mRNA expression analysis and literature review, 16 expression-related CASEs were found to be correlated with 22 SFs, where the CASE-SF network was constructed. Although a comprehensive analysis was conducted for constructing the colon cancer prognostic model, this research lacked wet experimental data to prove the conclusions. To fulfill our research, we plan to construct a sample library of colon cancer to evaluate the prognostic model in a cohort study manner.

## Figures and Tables

**Table 1:** Primer list.

Gene	Primers (5′-3′)	
tcf7	F	CTGACCTCTCTGGCTTCTACTC
	R	CAGAACCTAGCATCAAGGATGGG

slc13a3	F	CCATTGAGGAGTGGAACCTGCA
	R	GTGTTGCTCAGCCACATGGACA

lrrc36	F	CCAAGAGGTCACTAAGCCCATC
	R	ACCGTGCAAACTACCCAGGTCA

sult1a1	F	GGAGTTCATGGACCACAGCATC
	R	CCTGCCATCTTCTCCGCATAGT

cxcl12	F	CTCAACACTCCAAACTGTGCCC
	R	CTCCAGGTACTCCTGAATCCAC

*β*-Actin	F	CACCATTGGCAATGAGCGGTTC
	R	AGGTCTTTGCGGATGTCCACGT

## Data Availability

No data were used to support this study.

## Supplementary Materials

Supplementary Materials
*Table S1.* Splicing factors collected from former studies. *Table S2.* Univariate Cox analysis results. *Table S3.* Expression status of the 5 parental genes.
