# Supplementary material for: Screening Gene Expression-Related Alternative Splicing Event Signature for Colon Cancer Prognostic Prediction
Source: J Oncol. 2022 Jan 27;2022:9952438. doi: 10.1155/2022/9952438 (PMC8813276; doi:10.1155/2022/9952438)
Supplement: Supplementary Materials — Table S1. Splicing factors collected from former studies. Table S2. Univariate Cox analysis results. Table S3. Expression status of the 5 parental genes. [file 9952438.f1.zip › 9952438.f1/Table S1 (1).pdf]

9G8
CUG-BP1
DAZAP1
ESRP1
ESRP2
ETR-3
FMRP
Fox-1
Fox-2
hnRNP A0
hnRNP A1
hnRNP A2/B1
hnRNP A3
hnRNP C
hnRNP C1
hnRNP C2
hnRNP D
hnRNP D0
hnRNP DL
hnRNP E1
hnRNP E2
hnRNP F
hnRNP G
hnRNP H1
hnRNP H2
hnRNP H3
hnRNP I (PTB)
hnRNP J
hnRNP K
hnRNP L
hnRNP LL
hnRNP M
hnRNP P (TLS)
hnRNP Q
hnRNP U
HTra2alpha
HTra2beta1
HuB
HuC
HuD
HuR
KSRP
MBNL1
Nova-1
Nova-2
nPTB
PSF
QKI
RBM25
RBM4
RBM5
Sam68
SAP155
SC35
SF1
SF2/ASF
SLM-1
SLM-2

SRm160
SRp20
SRp30c
SRp38
SRp40
SRp54
SRp55
SRp75
TDP43
TIA-1
TIAL1
YB-1
ZRANB2
