# Supplementary material for: Screening Gene Expression-Related Alternative Splicing Event Signature for Colon Cancer Prognostic Prediction
Source: J Oncol. 2022 Jan 27;2022:9952438. doi: 10.1155/2022/9952438 (PMC8813276; doi:10.1155/2022/9952438)
Supplement: Supplementary Materials — Table S1. Splicing factors collected from former studies. Table S2. Univariate Cox analysis results. Table S3. Expression status of the 5 parental genes. [file 9952438.f1.zip › 9952438.f1/Table S2 (1).pdf]

gene	HR	HR.95L	HR.95H	pvalue
SULT1A1_	75.61392	7.012866	815.2822	0.000363
CXCL12_A	0.016309	0.001012	0.26291	0.00371
TCF7_ES_7	10.76294	1.368027	84.6774	0.023963
LRRC36_A	4.811496	1.186336	19.51428	0.027867
SLC13A3_	0.373364	0.141553	0.984798	0.04649
