# Supplementary material for: Screening Gene Expression-Related Alternative Splicing Event Signature for Colon Cancer Prognostic Prediction
Source: J Oncol. 2022 Jan 27;2022:9952438. doi: 10.1155/2022/9952438 (PMC8813276; doi:10.1155/2022/9952438)
Supplement: Supplementary Materials — Table S1. Splicing factors collected from former studies. Table S2. Univariate Cox analysis results. Table S3. Expression status of the 5 parental genes. [file 9952438.f1.zip › 9952438.f1/Table S3.pdf]

id	CXCL12	SULT1A1	SLC13A3	TCF7	LRRC36
TCGA-AA-3531-11A-01R-A32Z-07	3978.648	5899.929	15.0901	382.2564	8.356926
TCGA-A6-2678-11A-01R-A32Z-07	3603.139	2333.941	11.38439	377.4783	9.675378
TCGA-AA-3514-11A-01R-A32Z-07	1820.603	4229.106	1.295407	522.2504	5.617776
TCGA-A6-2679-11A-01R-A32Z-07	3465.265	5541.48	7.251975	453.754	12.49428
TCGA-AA-3660-11A-01R-1723-07	3303.554	2445.772	15.2257	403.6527	21.91902
TCGA-A6-2686-11A-01R-A32Z-07	3323.155	3264.867	13.93472	383.0321	11.6151
TCGA-AA-3712-11A-01R-1723-07	3914.107	2180.241	10.03453	376.2172	18.8893
TCGA-A6-2683-11A-01R-A32Z-07	3048.676	2404.64	4.477511	518.1679	11.64533
TCGA-AA-3534-11A-01R-A32Z-07	3835.642	2161.344	21.03616	844.3006	11.14404
TCGA-AA-3516-11A-01R-A32Z-07	2424.999	3081.463	11.2775	314.58	6.993825
TCGA-A6-2675-11A-01R-1723-07	7986.621	1615.37	29.73626	364.2121	8.422725
TCGA-AA-3517-11A-01R-A32Z-07	4065.606	3548.939	24.54052	1096.596	9.003232
TCGA-AA-3520-11A-01R-A32Z-07	3850.263	2433.263	14.81561	542.3453	11.21123
TCGA-A6-2685-11A-01R-A32Z-07	2283.698	4869.926	4.553922	439.8222	35.65383
TCGA-AA-3655-11A-01R-1723-07	4048.815	3839.46	14.24394	575.4624	18.65884
TCGA-A6-2682-11A-01R-A32Z-07	2260.613	2520.358	9.059838	457.2565	9.722554
TCGA-A6-2680-11A-01R-A32Z-07	3256.553	5295.777	5.775452	808.6949	16.15138
TCGA-AZ-6601-11A-01R-1774-07	2967.11	2426.637	18.53728	574.6206	12.57674
TCGA-AA-3489-11A-01R-1839-07	6760.749	1057.201	13.53236	413.6151	11.95017
TCGA-AA-3525-11A-01R-A32Z-07	2992.817	4896.764	11.8047	784.6961	7.039834
TCGA-AA-3522-11A-01R-A32Z-07	3894.845	2891.875	4.33576	436.2471	10.06528
TCGA-AA-3518-11A-01R-1672-07	4234.763	4352.012	14.58705	365.8844	10.38889
TCGA-AZ-6603-11A-02R-1839-07	14326.59	340.1713	28.92917	399.3634	15.16835
TCGA-AA-3496-11A-01R-1839-07	8934.47	3625.019	31.53819	277.8786	8.743507
TCGA-AZ-6605-11A-01R-1839-07	10805.43	513.0594	69.40468	144.6053	4.507394
TCGA-AA-3662-11A-01R-1723-07	5974.258	2652.266	55.90346	313.7327	19.07698
TCGA-AA-3511-11A-01R-1839-07	3980.145	2680.066	13.36469	1164.759	29.2079
TCGA-AA-3713-11A-01R-1723-07	3684.464	3651.782	6.828312	307.4212	18.98594
TCGA-AZ-6600-11A-01R-1774-07	4608.141	1516.218	14.78851	471.6595	12.32639
TCGA-AA-3697-11A-01R-1723-07	3743.242	1183.049	4.229974	512.755	17.73358
TCGA-F4-6704-11A-01R-1839-07	3527.814	789.6118	24.78138	355.817	15.5027
TCGA-A6-2671-11A-01R-A32Z-07	3314.878	2722.287	17.09159	280.0108	9.408206
TCGA-AA-3663-11A-01R-1723-07	7075.807	1749.586	20.83391	582.9319	13.8746
TCGA-AZ-6599-11A-01R-1774-07	7262.193	1361.182	10.55163	329.5387	14.44234
TCGA-A6-5665-11A-01R-1653-07	3363.513	3948.757	10.91035	500.4222	5.430902
TCGA-A6-5662-11A-01R-1653-07	6481.075	1571.097	22.56508	1199.753	11.22792
TCGA-AZ-6598-11A-01R-1774-07	4432.089	1726.748	31.39543	410.2207	18.92289
TCGA-A6-5667-11A-01R-1723-07	2335.52	4581.05	14.89462	480.8997	13.50149
TCGA-A6-2684-11A-01R-A32Z-07	2913.122	6824.789	5.302858	402.755	8.432815
TCGA-A6-5659-11A-01R-1653-07	1974.869	2646.925	8.522121	293.0069	7.117268
TCGA-AA-3527-11A-01R-A32Z-07	3401.165	4182.83	10.09869	370.0282	7.002566
TCGA-G4-6310-01A-11R-1723-07	1635.081	459.4883	48.69178	2125.915	86.8495
TCGA-AA-3866-01A-01R-1022-07	684.213	556.5552	358.0291	1995.556	196.9266
TCGA-F4-6806-01A-11R-1839-07	315.5037	910.4509	57.09188	2327.71	127.203
TCGA-AY-A8YK-01A-11R-A41B-07	409.4788	786.455	1439.952	2435.562	608.6736
TCGA-AA-3852-01A-01R-0905-07	1082.683	850.9728	5.813855	1146.586	99.82037
TCGA-D5-5539-01A-01R-1653-07	880.3956	430.3564	227.2021	443.7003	244.2889
TCGA-AZ-6601-01A-11R-1774-07	1153.856	565.0258	14.04321	1587.523	22.07399
TCGA-AA-3994-01A-01R-1113-07	396.1434	105.3673	34.26405	2131.267	72.96244
TCGA-G4-6307-01A-11R-1723-07	501.2928	549.9689	89.50901	2345.697	116.4707
TCGA-CM-4747-01A-01R-1410-07	166.0962	475.8779	102.4954	1640.119	79.39305
TCGA-NH-A50V-01A-11R-A28H-07	510.2371	757.8799	13.24916	1795.165	70.8396
TCGA-A6-5667-01A-21R-1723-07	493.6295	387.9025	200.5349	2686.025	24.48757
TCGA-NH-A5IV-01A-42R-A37K-07	441.2051	930.6334	35.84666	451.1328	20.2994
TCGA-AA-3667-01A-01R-0905-07	328.9243	721.4188	35.49204	3755.206	137.5334
TCGA-QG-A5Z2-01A-11R-A28H-07	114.0717	510.9565	1.762012	1054.781	31.54698
TCGA-AA-A01X-01A-21R-A083-07	308.672	819.6572	35.93302	4570.704	327.8968

TCGA-AY-6197-01A-11R-1723-07	234.0989	651.0607	4.878983	1194.067	5.498961
TCGA-AA-3517-01A-01R-0821-07	357.7445	1017.297	1855.157	2723.893	125.3477
TCGA-AA-3968-01A-01R-1022-07	850.8044	434.0527	317.6747	1739.96	106.4504
TCGA-AA-3713-01A-21R-1723-07	406.7833	788.2806	9.492596	651.6084	66.02261
TCGA-AA-3527-01A-01R-0821-07	342.8422	2083.587	182.307	1570.596	15.58393
TCGA-AA-3673-01A-01R-0905-07	402.4671	567.1477	139.9981	2111.085	308.7548
TCGA-AA-3856-01A-01R-0905-07	461.2398	497.7542	230.6358	3184.849	77.67993
TCGA-CM-6676-01A-11R-1839-07	581.7521	779.2861	167.3913	3084.742	66.73434
TCGA-A6-2679-01A-02R-1410-07	195.5087	1467.861	107.6926	1620.179	168.8472
TCGA-DM-A28M-01A-12R-A16W-07	268.5252	520.6954	0.481818	2932.915	144.5867
TCGA-AA-A010-01A-01R-A089-07	454.6034	701.8583	14.74432	1356.589	58.21229
TCGA-AA-3697-01A-01R-1723-07	492.3221	490.4437	761.6489	1987.255	51.74732
TCGA-AA-3532-01A-01R-0821-07	596.1652	582.31	526.807	1633.661	125.792
TCGA-AZ-6606-01A-11R-1839-07	276.6597	801.3995	18.78408	1777.312	37.05436
TCGA-A6-2674-01A-02R-0821-07	4756.174	150.3299	29.35816	368.069	72.82797
TCGA-CA-6716-01A-11R-1839-07	452.8809	3046.607	26.91499	2602.035	115.4464
TCGA-NH-A6GC-01A-12R-A41B-07	1042.057	797.4098	15.9254	1295.855	511.6105
TCGA-AZ-6607-01A-11R-1839-07	1315.573	275.2928	32.33914	536.2315	6.248105
TCGA-AZ-4308-01A-01R-1410-07	275.0866	627.7712	630.8144	1788.941	230.8965
TCGA-AA-3524-01A-02R-0821-07	195.2335	620.1656	510.0953	1592.693	13.112
TCGA-AA-3848-01A-01R-0905-07	387.2712	915.5196	749.9022	1631.467	200.0745
TCGA-CM-5861-01A-01R-1653-07	302.0885	303.2621	1.259278	895.4641	7.169453
TCGA-A6-6138-01A-11R-1774-07	566.3868	299.634	113.5506	2862.129	48.08411
TCGA-AA-A01G-01A-01R-A002-07	372.0491	548.9626	93.32648	2261.771	40.71827
TCGA-AM-5820-01A-01R-1653-07	443.0424	290.994	9.48789	858.5017	74.5803
TCGA-AA-3492-01A-01R-1410-07	398.3619	3061.626	24.49781	351.6957	14.83261
TCGA-AA-3819-01A-01R-0905-07	333.7405	887.4227	158.7928	2765.433	114.728
TCGA-A6-2678-01A-01R-0821-07	400.922	344.4791	16.20387	2133.294	179.6617
TCGA-AA-3521-01A-01R-0821-07	272.3637	523.4219	60.48842	3251.277	110.8301
TCGA-DM-A1DA-01A-11R-A155-07	239.9477	397.2275	7.423177	2388.821	58.19636
TCGA-AA-3864-01A-01R-1022-07	508.3375	457.6441	63.21519	307.0484	42.65571
TCGA-F4-6855-01A-11R-1928-07	2360.96	366.0518	460.5065	1056.313	101.2443
TCGA-F4-6856-01A-11R-1928-07	429.4521	1172.257	4.463906	297.0013	88.34525
TCGA-AA-A02H-01A-01R-A089-07	429.3856	571.1977	201.0304	3458.457	100.84
TCGA-AA-A022-01A-21R-A16W-07	102.7382	342.7407	23.21347	703.645	3.309702
TCGA-AA-3844-01A-01R-1022-07	565.163	903.8602	50.53378	2975.417	427.2627
TCGA-AA-3952-01A-01R-1022-07	568.6368	9033.646	118.8177	1382.343	761.3488
TCGA-D5-5537-01A-21R-1928-07	244.4093	680.6884	527.5029	4590.433	210.136
TCGA-AA-3837-01A-01R-0905-07	323.2976	243.1415	54.15532	1886.486	42.51887
TCGA-AA-3511-01A-21R-1839-07	453.0433	949.5816	449.4759	2947.436	197.8786
TCGA-D5-6922-01A-11R-1928-07	926.2895	391.3753	483.6747	1101.459	70.84885
TCGA-AA-3556-01A-01R-0821-07	462.4501	161.12	19.28043	902.9687	90.12591
TCGA-CM-6674-01A-11R-1839-07	566.3672	566.3819	15.93451	1723.656	21.82907
TCGA-AA-3939-01A-01R-1022-07	250.3647	612.0776	312.5046	1055.19	167.122
TCGA-AA-A00K-01A-02R-A002-07	497.4701	478.5042	261.6802	1739.512	27.91685
TCGA-DM-A28A-01A-21R-A32Y-07	318.1445	753.8735	76.49977	1469.558	149.2077
TCGA-CK-5914-01A-11R-1653-07	494.8043	1002.166	22.84205	2810.875	89.63995
TCGA-G4-6320-01A-11R-1723-07	154.8954	106.4226	6.629459	1466.524	72.21352
TCGA-A6-A5ZU-01A-11R-A28H-07	798.039	199.0222	1932.203	2377.942	2667.738
TCGA-AA-3495-01A-01R-1410-07	440.8727	814.4852	101.1149	1685.042	91.95941
TCGA-QL-A97D-01A-12R-A41B-07	516.6872	1348.531	108.1088	1321.567	403.6541
TCGA-CM-5868-01A-01R-1653-07	531.2559	592.2743	107.2169	2658.217	165.2514
TCGA-AA-3534-01A-01R-0821-07	901.6823	452.5337	97.09789	2580.959	98.4856
TCGA-AA-3693-01A-01R-0905-07	414.1921	299.5281	18.98449	3296.393	95.77671
TCGA-AA-A02J-01A-01R-A00A-07	262.6532	644.3695	849.2701	3059	293.9865
TCGA-D5-5541-01A-01R-1653-07	1144.493	254.2073	179.6449	2001.551	64.99691
TCGA-AA-3860-01A-02R-0905-07	247.5706	192.7794	92.07458	1880.862	103.7889
TCGA-A6-5657-01A-01R-A32Z-07	1602.876	219.5296	24.27412	1671.142	32.60474

TCGA-AA-3812-01A-01R-0905-07	638.8827	665.0832	71.66589	1189.351	27.52143
TCGA-DM-A1D4-01A-21R-A155-07	489.2466	1322.116	93.62438	1613.668	113.4177
TCGA-AA-3854-01A-01R-0905-07	277.5707	176.706	4.133381	1927.258	87.71191
TCGA-CK-4950-01A-01R-1723-07	438.6813	729.5667	23.496	1095.962	13.68084
TCGA-D5-6541-01A-11R-1723-07	1302.951	662.74	518.423	1793.21	99.79246
TCGA-QG-A5YV-01A-11R-A28H-07	271.2904	852.0767	55.8098	2273.499	191.5709
TCGA-AD-6901-01A-11R-1928-07	4500.371	455.3065	147.6791	2130.641	130.5141
TCGA-AA-3526-01A-02R-A32Z-07	501.9756	605.3934	27.16094	2119.721	113.9551
TCGA-DM-A1HA-01A-11R-A155-07	118.0452	93.1186	10.68708	4041.09	260.4223
TCGA-G4-6323-01A-11R-1723-07	291.4625	429.5118	4.49775	2457.404	331.5887
TCGA-AA-3811-01A-01R-1022-07	433.0654	478.5031	576.3871	458.0767	28.13302
TCGA-AA-3831-01A-01R-0905-07	660.9151	1055.497	138.8546	1447.916	30.40939
TCGA-AY-A54L-01A-11R-A28H-07	196.1944	919.6072	138.4978	2751.363	1500.601
TCGA-AD-6963-01A-11R-1928-07	541.8345	291.0365	104.0499	1930.661	104.7149
TCGA-A6-A566-01A-11R-A28H-07	3135.952	884.7224	77.47791	1067.993	73.84186
TCGA-AA-3518-01A-02R-0826-07	358.9362	1005.965	18.91772	1166.314	29.80002
TCGA-CM-5863-01A-21R-1839-07	1449.428	398.716	24.19483	1502.854	36.36608
TCGA-A6-5666-01A-01R-1653-07	415.7337	526.1468	503.8493	3256.816	333.8699
TCGA-DM-A280-01A-12R-A16W-07	631.3295	794.4542	2.85995	2066.722	12.45171
TCGA-AA-3973-01A-01R-1022-07	415.2064	560.716	1049.516	4274.359	128.4694
TCGA-CM-4748-01A-01R-1410-07	701.1205	205.7102	358.1476	1544.094	77.84373
TCGA-D5-6530-01A-11R-1723-07	459.482	688.5685	13.81217	1079.199	19.99245
TCGA-A6-6137-01A-11R-1774-07	299.1805	287.7525	596.8929	1840.416	98.11822
TCGA-DM-A1D6-01A-21R-A155-07	144.5326	560.0753	12.64577	730.5977	179.7191
TCGA-D5-6923-01A-11R-A32Z-07	1222.662	212.6437	773.9048	1591.192	28.19276
TCGA-A6-2681-01A-01R-1410-07	1076.861	288.475	116.0169	1345.618	173.7298
TCGA-F4-6463-01A-11R-1723-07	1705.93	279.1005	34.39296	3524.452	23.59629
TCGA-AZ-6599-01A-11R-1774-07	1439.308	432.9407	4.295996	1623.885	234.4293
TCGA-D5-6532-01A-11R-1723-07	468.9034	1052.433	3318.857	3243.719	270.4114
TCGA-AA-3489-01A-21R-1839-07	4932.52	345.755	214.9963	2235.91	53.16131
TCGA-CM-6171-01A-11R-1653-07	575.9572	230.1208	4.665363	1161.597	23.20103
TCGA-AA-A00R-01A-01R-A002-07	449.3603	413.8003	16.46886	241.0522	5.149171
TCGA-DM-A28C-01A-11R-A32Y-07	470.0706	228.4353	78.69471	2745.9	177.2748
TCGA-AA-A01T-01A-21R-A16W-07	315.6798	5701.524	236.6385	1944.569	385.5183
TCGA-AA-A02E-01A-01R-A00A-07	815.8355	875.279	206.3295	2683.973	65.02884
TCGA-CM-5860-01A-01R-1653-07	554.7673	416.2781	255.0216	2349.825	93.74417
TCGA-A6-6141-01A-11R-1774-07	242.0244	1558.913	13.34564	1495.333	294.0024
TCGA-AA-A00D-01A-01R-A002-07	848.1659	419.3965	450.1166	1902.693	37.99476
TCGA-G4-6628-01A-11R-1839-07	1812.842	457.8139	7.928351	372.2533	2.93918
TCGA-AA-3688-01A-01R-0905-07	479.9785	528.5387	996.19	2954.043	339.8262
TCGA-D5-6929-01A-31R-1928-07	674.9848	924.4644	538.1537	1874.139	478.2352
TCGA-AA-3984-01A-02R-1022-07	361.6933	559.9093	47.40623	1192.65	16.37607
TCGA-A6-5664-01A-21R-1839-07	2687.7	332.7149	24.50513	2203.295	47.86056
TCGA-AM-5821-01A-01R-1653-07	191.1653	106.9126	13.40101	630.1189	3.294298
TCGA-AA-3488-01A-01R-1410-07	541.1102	251.5886	809.2269	1959.944	188.6095
TCGA-AA-3956-01A-02R-1022-07	642.4456	612.3899	193.0919	2256.174	185.9443
TCGA-G4-6298-01A-11R-1723-07	227.905	467.3013	14.84329	2604.15	91.14863
TCGA-DM-A28F-01A-11R-A32Y-07	207.4953	1013.368	8.092941	742.0857	190.6924
TCGA-AA-A00J-01A-02R-A002-07	165.7064	322.1513	240.0977	1374.325	85.12623
TCGA-A6-6142-01A-11R-1774-07	2320.762	952.9377	1133.189	3404.779	96.46872
TCGA-G4-6304-01A-11R-1928-07	97.77851	671.8207	4.363972	1214.108	23.90793
TCGA-A6-2674-01A-02R-A278-07	2467.305	86.05429	27.17825	310.36	62.95189
TCGA-5M-AAT5-01A-21R-A41B-07	192.0761	1620.805	99.85608	2756.717	140.2646
TCGA-CA-6718-01A-11R-1839-07	606.5554	204.3989	15.89916	1460.665	9.776186
TCGA-G4-6627-01A-11R-1774-07	1823.643	340.4528	395.4692	1802.594	41.17542
TCGA-G4-6625-01A-21R-1774-07	1852.87	505.4858	90.71806	2518.651	73.51043
TCGA-A6-5656-01A-21R-1839-07	231.2308	833.5971	2477.486	4198.321	559.87
TCGA-AA-3710-01A-01R-1022-07	396.1887	328.7746	19.39172	389.36	9.018563

TCGA-G4-6306-01A-11R-1774-07	233.3526	2143.826	108.7943	3268.842	168.4849
TCGA-F4-6854-01A-11R-1928-07	791.3322	410.1787	355.7787	3901.644	88.91237
TCGA-AD-6890-01A-11R-1928-07	374.4822	394.4871	50.85611	2148.054	6.317329
TCGA-A6-3807-01A-01R-1022-07	237.1551	1385.676	236.9045	1962.809	78.5829
TCGA-A6-6782-01A-11R-1839-07	2259.036	1147.797	793.8167	3170.994	217.8465
TCGA-AA-A01C-01A-01R-A00A-07	778.8227	610.264	456.8393	2209.303	184.3795
TCGA-AA-3855-01A-01R-1022-07	853.6682	2604.294	12.25063	1606.742	80.66198
TCGA-A6-6653-01A-11R-1774-07	617.5348	467.0137	8.909072	1019.951	4.817849
TCGA-AA-A01R-01A-21R-A083-07	101.0773	770.5185	18.88843	1015.746	1.80172
TCGA-A6-2671-01A-01R-1410-07	1984.088	346.9799	78.05935	3728.66	22.76171
TCGA-AA-3663-01A-01R-1723-07	316.835	391.3537	2.646492	1236.981	60.99619
TCGA-A6-2672-01A-01R-0826-07	547.3837	792.7495	18.45565	1237.121	10.88117
TCGA-G4-6294-01A-11R-1774-07	137.4019	531.4482	697.883	2398.483	269.8449
TCGA-A6-2676-01A-01R-0826-07	338.9801	735.4368	752.4654	66.43045	0.259642
TCGA-AA-3549-01A-02R-0821-07	396.5704	871.7859	1317.146	1924.565	289.5145
TCGA-AA-3664-01A-01R-0905-07	273.9627	553.3456	59.48992	1409.386	159.7435
TCGA-AZ-4313-01A-01R-1410-07	217.4561	543.1196	43.68734	1417.497	67.5569
TCGA-AA-3530-01A-01R-1022-07	792.4607	1348.184	53.08494	1860.794	102.8434
TCGA-AA-3877-01A-01R-1022-07	1097.155	709.4269	28.42595	1155.251	22.78898
TCGA-D5-6535-01A-11R-1723-07	532.113	171.1066	19.05599	897.4557	50.8023
TCGA-CM-6678-01A-11R-1839-07	528.8436	3217.514	113.3031	1604.115	152.6105
TCGA-CM-4746-01A-01R-1410-07	562.9298	1585.786	6.53065	615.594	259.3481
TCGA-A6-6652-01A-11R-1774-07	311.1518	437.1421	122.2278	4108.606	105.1247
TCGA-D5-6534-01A-21R-1928-07	3361.407	277.3775	43.84567	1512.902	21.53725
TCGA-AA-3496-01A-21R-1839-07	639.7514	724.6646	2319.627	1867.972	73.76145
TCGA-AA-A02K-01A-03R-A32Y-07	145.1647	1609.491	1330.529	1523.891	120.0579
TCGA-AA-3685-01A-02R-A32Z-07	395.9071	718.4277	28.442	2762.292	116.4695
TCGA-AA-3851-01A-01R-1022-07	385.9011	653.4276	6.292589	715.6484	96.94578
TCGA-DM-A282-01A-12R-A16W-07	314.1292	1027.675	168.4845	3122.136	210.773
TCGA-AA-3543-01A-01R-0826-07	221.8812	339.8718	5.139982	1384.484	13.26494
TCGA-F4-6805-01A-11R-1839-07	1350.707	265.6278	21.91055	1405.155	25.16686
TCGA-AA-3821-01A-01R-1022-07	499.0091	1074.735	9.34859	438.4628	27.57092
TCGA-F4-6460-01A-11R-1774-07	887.9826	723.9273	1193.993	4366.005	362.0595
TCGA-AZ-5407-01A-01R-1723-07	302.6947	271.0253	3.694235	1502.448	32.56471
TCGA-NH-A6GA-01A-11R-A37K-07	245.8031	356.0836	12.31089	1638.29	9.398365
TCGA-D5-6537-01A-11R-1723-07	375.9175	1240.127	36.38953	2631.018	43.4266
TCGA-AZ-4684-01A-01R-1410-07	313.4031	1093.991	1048.28	1300.569	91.24898
TCGA-G4-6302-01A-11R-1723-07	4339.248	221.0613	190.6659	437.2762	4.84474
TCGA-AA-3862-01A-01R-1022-07	366.7305	344.901	563.8598	2726.514	184.9829
TCGA-A6-6781-01A-22R-A278-07	1264.641	287.3649	159.2946	363.7942	23.98929
TCGA-AA-A01Q-01A-01R-A002-07	224.1387	336.8409	10.37374	406.2175	112.997
TCGA-A6-5661-01A-01R-1653-07	392.0582	243.7871	5.448545	738.978	22.68495
TCGA-5M-AATE-01A-11R-A41B-07	253.3441	336.7222	506.6073	3208.631	337.677
TCGA-DM-A1D0-01A-11R-A155-07	196.2932	969.689	47.29331	2880.241	110.8535
TCGA-AA-3681-01A-01R-0905-07	491.1587	421.5901	21.74132	3875.552	25.6254
TCGA-D5-5538-01A-01R-1653-07	870.9234	552.7896	513.4841	1313.824	150.7845
TCGA-AA-3870-01A-01R-1022-07	659.4819	150.356	8.555328	2246.698	30.01997
TCGA-CM-6679-01A-11R-1839-07	961.2668	1372.944	318.5203	2147.69	214.9748
TCGA-T9-A92H-01A-11R-A37K-07	379.0403	767.8176	161.4991	2155.909	134.6774
TCGA-A6-3809-01A-01R-1022-07	577.8114	295.5074	14.89383	418.6954	2.182997
TCGA-AA-3818-01A-01R-0905-07	434.9429	374.2672	31.74323	1776.042	385.0743
TCGA-CA-5254-01A-21R-1839-07	340.2716	262.4591	6.820866	1457.695	61.82093
TCGA-AA-3672-01A-01R-0905-07	289.5225	552.977	71.57228	646.8908	1.499107
TCGA-AD-5900-01A-11R-1653-07	1033.103	123.2528	6.142487	1450.75	23.30572
TCGA-CA-6719-01A-11R-1839-07	481.0184	135.0959	238.4756	2135.814	37.8084
TCGA-A6-6654-01A-21R-1839-07	1214.688	198.2527	112.3614	1439.518	37.11159
TCGA-CM-6161-01A-11R-1653-07	2217.752	708.5433	110.9981	3098.783	43.04468
TCGA-AA-3814-01A-01R-0905-07	593.3788	476.3469	282.5815	1785.291	233.8805

TCGA-AA-3660-01A-01R-1723-07	441.6725	416.6227	1121.256	2331.396	116.6599
TCGA-CM-6166-01A-11R-1653-07	610.0936	194.0732	148.4558	2317.034	133.2872
TCGA-AA-A01P-01A-21R-A083-07	544.5821	178.4482	15.51644	878.8372	23.88547
TCGA-DM-A28G-01A-11R-A16W-07	499.3353	345.8944	39.69819	1667.247	99.25884
TCGA-CA-5256-01A-01R-1410-07	207.507	1836.323	123.9664	2686.77	171.75
TCGA-CM-6675-01A-11R-1839-07	16.39137	749.2894	5.562472	3419.77	101.2083
TCGA-AA-A029-01A-01R-A00A-07	118.8665	298.3749	14.19883	1252.156	0.945685
TCGA-AA-3678-01A-01R-0905-07	554.7327	497.579	4536.611	2911.27	239.7311
TCGA-A6-6140-01A-11R-1774-07	365.0623	499.9944	4587.02	5601.023	98.82242
TCGA-CM-6169-01A-11R-1653-07	1400.174	202.7817	1287.738	2324.904	56.6347
TCGA-AA-3516-01A-02R-0826-07	291.0532	309.9416	8.94947	452.7733	8.970851
TCGA-AA-3552-01A-01R-0821-07	837.0562	666.791	481.7744	1799.489	48.50093
TCGA-D5-6931-01A-11R-1928-07	454.7681	382.7253	8.081122	1269.88	5.332019
TCGA-SS-A7HO-01A-21R-A37K-07	518.6059	1230.848	1130.684	2438.638	123.9795
TCGA-AZ-6605-01A-11R-1839-07	2070.136	428.4191	100.8376	1576.561	70.00908
TCGA-CA-6717-01A-11R-1839-07	1325.346	485.2135	19.74035	1449.01	6.661204
TCGA-NH-A8F7-01A-11R-A41B-07	315.6528	785.0688	2351.078	6189.164	568.5433
TCGA-AD-6965-01A-11R-1928-07	409.5661	537.218	24.49116	1658.793	47.6302
TCGA-A6-2685-01A-01R-1410-07	3511.351	1981.84	809.2207	1864.968	301.6146
TCGA-AA-3712-01A-21R-1723-07	515.6361	338.5005	178.8094	3543.497	124.2283
TCGA-AA-3562-01A-02R-0821-07	532.8732	2319.595	67.0762	1814.581	110.7361
TCGA-AA-3846-01A-01R-1022-07	294.917	604.5135	12.34309	2470.547	301.6552
TCGA-A6-A565-01A-31R-A28H-07	1685.553	1855.769	22.80823	1680.518	74.42407
TCGA-AU-3779-01A-01R-1723-07	378.1032	1777.235	37.45814	3135.007	236.8266
TCGA-A6-2684-01A-01R-A278-07	948.746	466.6408	18.0339	641.7871	85.68931
TCGA-AA-3561-01A-01R-0821-07	220.3746	699.8184	171.7912	1280.313	231.6861
TCGA-CM-5341-01A-01R-1410-07	544.0816	566.0876	12.90008	355.0371	110.4819
TCGA-A6-2683-01A-01R-0821-07	173.6492	351.1166	713.8577	2276.048	273.5376
TCGA-AA-A004-01A-01R-A00A-07	1500.557	611.7248	47.78293	1416.038	80.72258
TCGA-AA-3529-01A-02R-0821-07	645.8124	497.3214	226.8706	1285.317	37.81846
TCGA-AA-3861-01A-01R-1022-07	497.0183	98.28378	6.454135	2080.434	103.973
TCGA-AY-A69D-01A-11R-A37K-07	611.1222	1568.381	151.316	1483.742	231.6636
TCGA-F4-6808-01A-11R-1839-07	276.2737	390.7133	2311.412	2504.471	107.9026
TCGA-CM-6162-01A-11R-1653-07	2413.295	243.5177	34.62728	365.0178	16.23533
TCGA-F4-6704-01A-11R-1839-07	1315.109	213.6155	1117.496	211.2631	73.40135
TCGA-AD-6888-01A-11R-1928-07	182.2136	367.933	405.3841	2808.717	144.4788
TCGA-A6-A56B-01A-31R-A28H-07	371.8254	266.1938	381.6865	1552.662	233.0568
TCGA-CM-4750-01A-01R-1410-07	202.3925	245.5692	99.02045	2169.089	61.95534
TCGA-A6-4105-01A-02R-1774-07	646.8806	215.7355	123.8309	1935.105	49.73155
TCGA-AA-3548-01A-01R-1873-07	262.3541	1743.381	55.66983	2184.684	217.0317
TCGA-AY-6386-01A-21R-1723-07	510.4623	1528.943	8.860631	1616.184	14.83665
TCGA-AY-4071-01A-01R-1113-07	280.0014	1158.557	1071.613	2818.636	204.2119
TCGA-F4-6569-01A-11R-1774-07	3593.617	875.1049	64.77837	2276.878	6.122219
TCGA-AA-3976-01A-01R-1022-07	546.4331	670.9619	1235.305	3538.659	161.4162
TCGA-A6-5656-01A-21R-A278-07	264.0486	431.3978	1798.185	1581.539	360.4931
TCGA-AD-6899-01A-11R-1928-07	656.0649	136.0932	30.93108	1439.504	55.11335
TCGA-A6-6651-01A-21R-1839-07	4555.676	443.286	294.7843	1913.034	32.59672
TCGA-AA-3977-01A-01R-1022-07	1028.922	220.8956	26.33727	1348.508	51.64445
TCGA-AA-A00Z-01A-01R-A002-07	519.8298	517.0876	68.04237	2545.563	114.0538
TCGA-NH-A50U-01A-33R-A37K-07	199.611	766.1067	43.86845	1819.801	147.9806
TCGA-D5-6928-01A-11R-1928-07	2176.255	244.5688	30.72699	1531.082	24.41715
TCGA-DM-A28K-01A-21R-A32Y-07	1741.51	609.0234	137.8853	862.1812	95.01065
TCGA-AY-6196-01A-11R-1723-07	7424.319	469.1695	89.79274	1871.926	73.32223
TCGA-CM-5344-01A-21R-1723-07	665.1173	693.1452	44.33379	527.2506	36.44519
TCGA-G4-6309-01A-21R-1839-07	276.3974	1142.259	409.8194	617.067	100.343
TCGA-CM-6165-01A-11R-1653-07	1040.577	637.9127	60.12311	1819.792	101.8241
TCGA-AY-5543-01A-01R-1653-07	387.5686	590.3739	171.6083	2466.631	489.0979
TCGA-CM-6167-01A-11R-1653-07	3652.19	267.8854	60.63255	1613.165	314.4717

TCGA-4T-AA8H-01A-11R-A41B-07	86.29519	1100.391	2.566664	1988.071	394.726
TCGA-AA-A00Q-01A-01R-A002-07	223.1721	456.5735	185.1937	764.4735	139.5238
TCGA-AZ-4614-01A-01R-1410-07	138.379	373.7422	163.7064	1046.188	19.95844
TCGA-A6-2675-01A-02R-1723-07	1246.427	712.6256	31.77776	2284.945	76.93351
TCGA-AA-A00U-01A-01R-A002-07	774.2198	127.4724	34.56574	1673.947	37.81726
TCGA-A6-6781-01A-22R-1928-07	2213.257	460.0092	208.1927	680.3729	24.8102
TCGA-CK-6748-01A-11R-1839-07	609.2549	689.4795	45.67224	4868.635	122.9153
TCGA-CA-5255-01A-11R-1839-07	67.05789	682.7683	5.319582	2094.854	80.49772
TCGA-CK-4952-01A-01R-1723-07	438.6533	90.98461	60.60261	1427.865	131.3915
TCGA-AZ-4315-01A-01R-1410-07	237.7739	1106.979	24.94908	1120.971	204.9486
TCGA-AA-3869-01A-01R-1022-07	750.8929	453.7075	293.1457	1543.662	76.91364
TCGA-G4-6626-01A-11R-1774-07	752.7238	321.1801	838.9274	2511.334	66.08223
TCGA-DM-A1D7-01A-11R-A155-07	917.3868	1704.631	83.49185	1731.444	159.4129
TCGA-AA-3531-01A-01R-0821-07	328.7658	2232.468	1297.16	3530.489	167.1158
TCGA-A6-5662-01A-01R-1653-07	390.9247	1118.391	512.9592	4362.479	159.9579
TCGA-CM-4744-01A-01R-A32Z-07	153.2521	300.7402	9.134976	2676.266	15.09608
TCGA-D5-5540-01A-01R-1653-07	595.7027	996.0643	40.20203	2106.069	222.2171
TCGA-AZ-6608-01A-11R-1839-07	295.4001	397.0821	112.8412	4789.677	253.6829
TCGA-AA-3684-01A-02R-0905-07	486.4336	1746.678	127.7929	1278.949	30.20344
TCGA-F4-6807-01A-11R-1839-07	2743.129	151.6838	348.608	1667.84	45.56168
TCGA-AA-3875-01A-01R-0905-07	873.6901	697.5306	114.2264	2351.215	97.52259
TCGA-G4-6297-01A-11R-1723-07	588.4221	361.4	164.9973	1781.111	55.57187
TCGA-A6-5660-01A-01R-1653-07	1450.505	370.5365	82.25571	1981.906	77.76539
TCGA-AA-A01D-01A-01R-A00A-07	1503.219	350.5106	185.3864	863.323	44.4741
TCGA-CM-5862-01A-01R-1653-07	439.4375	339.1987	1100.577	2153.624	254.1325
TCGA-CK-5916-01A-11R-1653-07	560.2132	161.4639	6.337162	1752.325	3.923557
TCGA-AA-3692-01A-01R-0905-07	460.0679	496.462	292.3608	1685.686	790.4492
TCGA-AA-A00O-01A-02R-A089-07	3765.131	386.1639	2342.035	2653.882	139.4159
TCGA-AA-A01I-01A-02R-A089-07	507.194	1197.385	754.9154	1764.558	234.4852
TCGA-AY-A71X-01A-12R-A37K-07	100.9237	385.2669	5.196932	2426.31	465.3924
TCGA-F4-6809-01A-11R-1839-07	2151.678	842.9996	176.2614	4285.819	202.1227
TCGA-G4-6321-01A-11R-1723-07	760.9492	1560.487	8.053864	984.5994	11.48267
TCGA-CM-6172-01A-11R-1653-07	1281.424	779.5676	6.587714	2001.447	203.7772
TCGA-AD-A5EJ-01A-11R-A28H-07	283.4806	422.3379	8.086795	921.6137	58.84269
TCGA-AA-3966-01A-01R-1113-07	493.0213	159.4313	26.47458	395.2372	20.20655
TCGA-AA-A00L-01A-01R-A002-07	432.4132	567.508	40.81141	3737.297	156.2856
TCGA-D5-6927-01A-21R-1928-07	523.555	252.9269	19.18479	309.0488	36.776
TCGA-D5-6536-01A-11R-1723-07	1369.213	630.0552	153.5733	3504.173	363.7097
TCGA-D5-6539-01A-11R-1723-07	594.8721	2854.602	7.160756	1259.788	63.31008
TCGA-A6-2686-01A-01R-A32Z-07	385.0471	383.1515	602.3002	557.8465	20.21061
TCGA-A6-3810-01A-01R-A278-07	843.9388	337.8003	156.9568	723.3955	104.9824
TCGA-F4-6461-01A-11R-1774-07	713.7807	297.7169	16.43681	664.855	107.8894
TCGA-A6-5659-01A-01R-A278-07	380.3918	115.7113	150.7033	484.2316	109.8313
TCGA-AD-6895-01A-11R-1928-07	637.8545	176.4429	11.6408	910.7646	15.3086
TCGA-A6-3810-01A-01R-1022-07	1486.663	515.2004	127.2378	1263.943	142.6272
TCGA-AA-3510-01A-01R-1410-07	660.5418	558.2798	45.68055	1464.482	52.75663
TCGA-CA-6715-01A-21R-1839-07	285.6923	1226.998	2388.035	4412.693	200.5099
TCGA-AA-3850-01A-01R-1022-07	1730.694	2728.084	361.2574	1486.561	14.25605
TCGA-AZ-4616-01A-21R-1839-07	329.9914	324.8995	12.98119	2324.719	89.54629
TCGA-AA-3715-01A-01R-0905-07	850.5785	290.3251	28.51074	348.3737	5.277524
TCGA-AA-3506-01A-01R-1410-07	504.7704	559.7306	85.74282	2811.867	82.10628
TCGA-AD-6964-01A-11R-1928-07	1360.719	439.4425	36.44844	1617.873	25.61001
TCGA-G4-6586-01A-11R-1774-07	149.1301	1035.194	2.711604	1034.24	30.41231
TCGA-AZ-6600-01A-11R-1774-07	1258.576	336.7918	23.68934	1252.743	196.1767
TCGA-AA-3675-01A-02R-0905-07	713.6545	947.6117	135.8369	1652.078	122.3528
TCGA-D5-6529-01A-11R-1774-07	916.6363	525.1299	36.41384	1619.773	42.73595
TCGA-4N-A93T-01A-11R-A37K-07	183.5339	1591.543	6.744914	1651.401	261.587
TCGA-A6-6780-01A-11R-1839-07	458.1712	633.6531	12.25094	235.5735	19.19578

TCGA-AA-3842-01A-01R-1022-07	193.7516	468.2844	150.4872	1774.097	79.97212
TCGA-AA-3542-01A-02R-1873-07	306.7476	722.4612	1872.495	1343.602	126.1566
TCGA-AA-3560-01A-01R-0821-07	279.6872	736.456	1103.988	1059.491	184.9404
TCGA-AA-3845-01A-01R-1022-07	348.57	521.0305	11.26361	381.5664	12.85299
TCGA-AA-A02F-01A-01R-A089-07	457.1088	198.7817	1692.856	3628.835	95.02803
TCGA-G4-6322-01A-11R-1723-07	1009.437	198.618	8.3488	805.4767	186.7293
TCGA-3L-AA1B-01A-11R-A37K-07	999.8384	447.4594	10.80409	2334.146	486.5979
TCGA-AZ-6603-01A-11R-1839-07	1133.794	236.3793	429.5274	2988.692	170.714
TCGA-AZ-5403-01A-01R-1653-07	629.1004	411.2691	499.3117	721.6644	147.2688
TCGA-AD-6548-01A-11R-1839-07	825.941	323.5822	29.32144	1005.385	6.880845
TCGA-AA-3554-01A-01R-0826-07	1367.972	287.9076	15.48481	770.3961	30.72124
TCGA-CK-5913-01A-11R-1653-07	544.4491	165.0139	8.71825	710.2256	15.05354
TCGA-AA-A01S-01A-21R-A083-07	302.2386	709.0983	913.1481	3485.936	91.34165
TCGA-AA-A02W-01A-01R-A00A-07	303.5021	333.7091	1864.071	2712.402	54.49449
TCGA-AA-A00F-01A-01R-A002-07	409.5496	778.8885	240.7261	1925.882	95.36139
TCGA-AA-A02Y-01A-43R-A32Y-07	73.30449	404.1488	176.9036	2520.51	231.2319
TCGA-AA-3525-01A-02R-0826-07	415.175	1268.501	19.69121	1156.191	3.521981
TCGA-A6-6650-01A-11R-A278-07	452.9657	1421.077	32.70936	985.7952	468.5595
TCGA-CM-4752-01A-01R-1410-07	455.5949	430.2727	332.0772	1208.206	298.2124
TCGA-A6-3809-01A-01R-A278-07	400.6631	263.8368	10.84479	262.4431	6.71571
TCGA-CM-6680-01A-11R-1839-07	853.774	349.9006	8.749956	1210.073	150.4871
TCGA-AD-A5EK-01A-11R-A28H-07	325.2732	581.2669	6.417508	3910.677	273.388
TCGA-A6-2682-01A-01R-1410-07	503.3347	392.704	204.1634	1236.367	42.90467
TCGA-CM-6164-01A-11R-1653-07	1033.094	298.9325	284.2407	2895.849	107.3723
TCGA-DM-A28H-01A-11R-A16W-07	556.0153	1993.335	53.30995	736.8048	45.32766
TCGA-D5-6898-01A-11R-1928-07	549.2527	223.1523	98.09031	1419.317	10.44302
TCGA-A6-3808-01A-01R-1022-07	2559.083	324.1289	22.76135	1755.677	45.19826
TCGA-A6-6780-01A-11R-A278-07	339.5715	340.6354	4.994459	203.8273	12.27199
TCGA-AA-3662-01A-01R-1723-07	1053.023	750.4833	374.8929	2247.141	103.1442
TCGA-CM-5864-01A-01R-1653-07	633.2536	289.2318	832.1345	2293.501	280.0775
TCGA-NH-A6GB-01A-11R-A37K-07	444.2128	602.6626	14.5665	2824.768	355.8105
TCGA-NH-A8F8-01A-72R-A41B-07	593.8456	447.5815	49.99203	2932.388	1002.495
TCGA-CM-6677-01A-11R-1839-07	621.2059	433.7468	440.7283	1886.553	7.975491
TCGA-AA-3949-01A-01R-1022-07	1065.794	159.1224	19.61341	428.6158	14.90787
TCGA-DM-A1D9-01A-11R-A155-07	160.0837	794.3438	69.66469	2645.791	183.4594
TCGA-D5-6930-01A-11R-1928-07	650.6258	145.3744	11.11944	1148.921	15.41664
TCGA-AA-3980-01A-02R-1022-07	352.6112	254.4145	14.66718	1458.848	124.2696
TCGA-CA-5797-01A-01R-1653-07	410.5981	446.3813	3414.873	2107.188	110.661
TCGA-AA-3494-01A-01R-1410-07	330.6377	376.6963	776.4449	1433.953	52.82096
TCGA-AA-3696-01A-01R-0905-07	325.4786	721.3634	41.92568	1666.413	233.9986
TCGA-AA-3679-01A-02R-0905-07	601.4809	1153.916	2983.652	2880.803	155.9585
TCGA-AA-A03J-01A-21R-A16W-07	168.6005	1549.323	1891.516	3292.466	233.6561
TCGA-F4-6703-01A-11R-1839-07	6219.574	217.9241	268.0951	858.3434	13.25922
TCGA-RU-A8FL-01A-11R-A37K-07	212.4508	441.0897	707.6579	2413.551	447.2185
TCGA-AA-3680-01A-01R-0905-07	736.6844	399.1962	17.88035	1732.249	73.78604
TCGA-A6-6650-01A-11R-1774-07	858.7684	1620.276	48.34251	2929.13	518.7269
TCGA-G4-6311-01A-11R-1723-07	427.9714	305.5456	30.29062	1804.222	32.62939
TCGA-AA-3815-01A-01R-1022-07	365.1736	357.0043	14.97847	2376.582	13.36566
TCGA-CK-4951-01A-01R-1410-07	495.6321	404.6194	12.77332	495.6817	8.985533
TCGA-AZ-4323-01A-21R-1839-07	2425.808	401.1381	45.55458	3410.922	25.34228
TCGA-A6-6648-01A-11R-1774-07	1536.716	412.8743	811.6871	1217.338	30.02482
TCGA-AA-A01Z-01A-11R-A083-07	254.5898	356.1058	568.4617	1823.223	146.3385
TCGA-WS-AB45-01A-11R-A41B-07	4564.627	404.5827	59.59178	160.9346	11.76211
TCGA-AA-3986-01A-02R-1022-07	1201.302	395.6788	52.66001	2213.168	28.44032
TCGA-G4-6293-01A-11R-1723-07	549.6994	519.5749	342.8785	2009.249	38.99623
TCGA-D5-6538-01A-11R-1723-07	285.5999	625.7865	342.1815	1983.288	167.2548
TCGA-AA-3941-01A-01R-1022-07	214.8058	1302.947	24.00025	2833.045	335.073
TCGA-G4-6317-01A-11R-1723-07	164.8442	682.9675	1547.546	2381.561	186.4475

TCGA-AA-3950-01A-02R-1022-07	983.5587	156.1561	20.58093	863.1701	15.09869
TCGA-AA-3971-01A-01R-1022-07	334.4607	1287.503	1706.989	3138.58	147.6135
TCGA-CK-6751-01A-11R-1839-07	773.0924	661.6176	27.96375	1533.339	7.116744
TCGA-F4-6570-01A-11R-1774-07	2274.718	237.7833	20.34772	870.9455	15.55527
TCGA-AA-3955-01A-02R-1022-07	258.6238	567.5764	211.3319	1542.598	141.3664
TCGA-D5-6533-01A-11R-1723-07	679.967	734.1271	48.8429	3469.72	125.7318
TCGA-CK-6746-01A-11R-1839-07	184.3296	243.1707	0	877.2364	2.05076
TCGA-DM-A288-01A-11R-A16W-07	68.46106	1842.269	2.87732	2799.978	95.4757
TCGA-A6-4107-01A-02R-1410-07	519.8865	1029.945	151.365	1433.853	547.0398
TCGA-QG-A5Z1-01A-11R-A28H-07	260.8953	657.5372	24.41637	2047.276	173.3697
TCGA-DM-A0XF-01A-11R-A155-07	318.4107	1836.779	581.3374	2291.329	228.4554
TCGA-AA-3947-01A-01R-1022-07	457.4184	271.3527	13.00975	592.8941	242.0412
TCGA-D5-6531-01A-11R-1723-07	281.7806	182.7976	4.653437	11160.89	40.27367
TCGA-AZ-4615-01A-01R-1410-07	462.7013	245.5741	14.27849	2016.145	13.00133
TCGA-A6-2684-01A-01R-1410-07	1134.261	629.2492	34.56908	1703.438	81.72975
TCGA-CM-4743-01A-01R-1723-07	297.1738	288.1685	10.00613	358.2094	18.01083
TCGA-AA-A01K-01A-01R-A00A-07	421.8952	541.8237	142.2972	2261.592	144.3971
TCGA-DM-A0X9-01A-11R-A155-07	499.193	308.5759	572.4913	2394.806	104.4371
TCGA-A6-5659-01A-01R-1653-07	676.2729	137.4036	182.5088	1862.373	114.0886
TCGA-AZ-6598-01A-11R-1774-07	232.1157	313.0736	707.3107	663.5519	71.11858
TCGA-AA-3519-01A-02R-0821-07	344.629	945.6361	661.2791	3973.483	194.8288
TCGA-DM-A1HB-01A-21R-A180-07	155.5161	330.2802	5.966742	1194.429	14.78479
TCGA-A6-A567-01A-31R-A28H-07	329.1321	471.0687	664.822	3671.292	124.1517
TCGA-AA-3970-01A-01R-1022-07	577.7862	642.9238	470.8214	1540.462	121.0268
TCGA-CA-5796-01A-01R-1653-07	343.3808	1617.942	2.263581	1480.779	80.88864
TCGA-5M-AAT6-01A-11R-A41B-07	2298.503	394.683	70.80238	549.2438	10.07898
TCGA-CM-6163-01A-11R-1653-07	389.8815	581.9055	66.76224	1255.231	539.5739
TCGA-AD-6889-01A-11R-1928-07	247.4851	128.2151	2.387012	472.0286	50.08563
TCGA-CM-4751-01A-02R-1839-07	473.1133	398.2519	70.76333	1779.658	8.931608
TCGA-AA-3982-01A-02R-1022-07	765.5584	388.3065	236.9868	1492.955	132.9777
TCGA-AA-A00A-01A-01R-A002-07	228.2021	381.6016	3.276269	1481.66	29.1512
TCGA-D5-6924-01A-11R-1928-07	1601.212	293.9081	339.9496	3474.69	137.1041
TCGA-AA-3502-01A-01R-1410-07	317.0107	235.9201	6.397314	2110.899	67.68316
TCGA-D5-7000-01A-11R-A32Z-07	596.2151	256.3666	12.67939	1248.739	37.85666
TCGA-D5-6540-01A-11R-1723-07	189.8288	175.328	16.53674	738.0222	10.73159
TCGA-D5-6932-01A-11R-1928-07	780.5212	640.4815	46.7189	1918.175	135.3161
TCGA-CK-5912-01A-11R-1653-07	1606.925	392.2366	4156.288	2784.534	382.564
TCGA-AA-3538-01A-01R-0821-07	489.4653	687.1404	486.7812	1861.154	63.94346
TCGA-AA-3930-01A-01R-1022-07	373.9166	871.1847	2.507745	1634.82	14.83915
TCGA-AA-A00W-01A-01R-A002-07	306.9546	568.3901	97.00491	3032.652	208.0333
TCGA-AA-3555-01A-01R-0821-07	472.4146	209.7638	13.04632	1556.082	54.27017
TCGA-AA-3655-01A-02R-1723-07	268.4201	521.645	4.768506	351.8553	91.76696
TCGA-AA-3989-01A-01R-1022-07	517.9253	1102.455	190.9719	2340.113	160.2341
TCGA-5M-AATA-01A-31R-A41B-07	1205.775	250.4735	24.84753	1688.236	131.7149
TCGA-5M-AAT4-01A-11R-A41B-07	433.5905	286.126	725.0107	785.305	197.1424
TCGA-AA-A02R-01A-01R-A00A-07	198.6852	252.26	8.908695	1128.546	3.109546
TCGA-A6-6649-01A-11R-1774-07	375.4692	660.4605	10.81675	1574.076	43.68176
TCGA-AY-4070-01A-01R-1113-07	566.9369	660.6551	722.7656	1180.015	16.50533
TCGA-G4-6299-01A-11R-1774-07	1164.228	525.5195	48.41712	979.1971	10.78329
TCGA-AA-A02O-01A-21R-A16W-07	228.5537	485.078	89.78886	1596.665	116.4746
TCGA-AA-3972-01A-01R-1022-07	420.4819	853.923	437.1145	4069.675	121.3833
TCGA-NH-A50T-01A-11R-A28H-07	256.448	489.6574	166.9134	2465.803	26.85279
TCGA-DM-A0XD-01A-12R-A155-07	74.64565	267.0048	150.1822	2131.464	292.3757
TCGA-AU-6004-01A-11R-1723-07	630.7858	416.9339	19.01428	662.9031	19.00401
TCGA-AA-3841-01A-01R-0905-07	263.0165	592.8089	620.1697	2026.134	160.1902
TCGA-DM-A28E-01A-11R-A32Y-07	127.9878	903.7057	193.5991	2550.461	152.2496
TCGA-AA-A00E-01A-01R-A002-07	379.11	459.5654	109.6566	833.1858	10.76046
TCGA-AA-3514-01A-02R-0821-07	1834.338	1780.49	48.29666	1296.59	32.98963

TCGA-AA-A01F-01A-01R-A002-07	172.5275	255.1465	851.1457	4221.917	335.6429
TCGA-G4-6315-01A-11R-1723-07	452.6769	670.5817	98.63834	3875.608	140.41
TCGA-F4-6459-01A-11R-1774-07	888.3815	590.7582	28.99711	524.899	46.04474
TCGA-D5-6926-01A-11R-1928-07	871.3036	323.2424	99.97724	1328.156	71.57399
TCGA-CK-6747-01A-11R-1839-07	439.4481	2336.189	22.612	1616.797	3.787162
TCGA-DM-A1D8-01A-11R-A155-07	362.8931	709.1528	9.799846	3091.022	137.082
TCGA-AA-3858-01A-01R-0905-07	583.274	341.2323	13.52567	1113.326	110.8257
TCGA-AA-3522-01A-01R-0821-07	269.7223	505.921	17.62648	1415.055	97.8516
TCGA-G4-6314-01A-11R-1723-07	1455.944	173.3766	27.99935	1496.936	48.69074
TCGA-AA-3544-01A-01R-1873-07	703.8916	606.9496	1017.83	3458.008	218.6172
TCGA-AA-3975-01A-01R-1022-07	570.8081	625.3634	749.9571	3564.827	237.5209
TCGA-AA-3666-01A-02R-0905-07	424.064	398.5756	233.0209	2304.059	214.2204
TCGA-AA-3872-01A-01R-1022-07	2026.627	700.5087	546.5264	2162.62	74.74283
TCGA-AA-3979-01A-01R-1022-07	155.8689	411.3009	325.4243	1877.727	98.57227
TCGA-CM-6168-01A-11R-1653-07	1448.955	703.0494	27.54987	879.5243	30.5005
TCGA-G4-6588-01A-11R-1774-07	374.6489	151.6574	10.84519	1380.785	10.05638
TCGA-CM-5348-01A-21R-1723-07	1288.05	82.23677	314.019	585.7992	23.75192
TCGA-AA-3553-01A-01R-0821-07	618.7343	222.0399	2529.794	1379.678	52.35631
TCGA-AA-3833-01A-01R-0905-07	589.1599	541.5534	15.08956	874.7543	13.46534
TCGA-G4-6295-01A-11R-1723-07	685.8398	203.3937	340.9887	2848.456	23.18094
TCGA-A6-2677-01A-01R-0821-07	1552.154	546.233	936.2981	1867.433	70.70981
TCGA-AA-3867-01A-01R-1022-07	1071.016	328.246	208.6324	2213.398	37.484
TCGA-AA-A00N-01A-02R-A00A-07	1483.205	1198.006	47.08329	972.8062	52.41794
TCGA-CK-5915-01A-11R-1653-07	7168.441	327.6379	121.0134	2590.22	18.09397
TCGA-AA-3520-01A-01R-0821-07	481.5461	980.8071	114.6207	1317.354	128.4184
TCGA-A6-5665-01A-01R-1653-07	98.36574	182.8039	6.542008	497.5825	10.23292
TCGA-G4-6303-01A-11R-1774-07	437.6758	274.1214	878.5475	2335.388	44.88678
TCGA-AA-A03F-01A-11R-A16W-07	283.1077	1496.674	4.372985	1973.721	47.59007
TCGA-DM-A1DB-01A-11R-A155-07	191.3849	1134.395	316.7516	2505.744	343.9304
TCGA-CM-6170-01A-11R-1653-07	502.1774	235.9331	15.13642	2915.886	140.4359
TCGA-A6-2680-01A-01R-1410-07	338.0115	591.2031	639.6179	2352.584	170.3002
TCGA-QG-A5YW-01A-11R-A28H-07	327.7056	297.4789	12.58082	986.4448	197.8165
TCGA-AA-A024-01A-02R-A00A-07	238.5888	867.0515	18.41742	1796.989	80.51108
TCGA-DM-A285-01A-11R-A16W-07	380.3968	330.091	375.0498	2518.047	19.04195
TCGA-QG-A5YX-01A-11R-A28H-07	286.8262	1520.903	2.596062	582.0218	76.58858
TCGA-AA-A017-01A-01R-A00A-07	182.167	585.7328	2485.516	4618.035	255.4911
TCGA-AA-3509-01A-01R-1410-07	743.6047	190.5477	2591.293	2779.941	327.673
TCGA-D5-6920-01A-11R-1928-07	637.6249	1358.286	11.24383	2063.077	16.04355
TCGA-CM-5349-01A-21R-1723-07	822.6067	841.83	270.9641	3094.686	98.27297
TCGA-AA-A01V-01A-23R-A083-07	209.4728	632.3367	30.89326	4441.225	75.85394
